# Effect of Nitrogen Doping on Tribological Properties of Ta_2_O_5_ Coatings Deposited by RF Magnetron Sputtering

**DOI:** 10.3390/ma15238291

**Published:** 2022-11-22

**Authors:** Rui Chao, Haichao Cai, Hang Li, Yujun Xue

**Affiliations:** 1 School of Mechatronics Engineering, Henan University of Science and Technology, Luoyang 471003, China; 2 Henan Key Laboratory for Machinery Design and Transmission System, Luoyang 471003, China; 3 Longmen Laboratory, Luoyang 471000, China

**Keywords:** magnetron sputtering, Ta_2_O_5_, TaN, friction, wear, surface morphology

## Abstract

Ta_2_O_5_ was deposited on quartz glass and Si substrates as a protective coating. The inherent RF magnetron sputtering power of 140 W was maintained during the deposition process. During the deposition process, amounts of 5%, 10%, and 15% of N_2_ were injected, and the total sputtering gas (N_2_+Ar) flow was kept at 40 sccm. The microstructure and surface morphology of the coatings were characterized, and the friction and wear experiments of the coatings were carried out. The results show that the coatings’ surface is smooth and the main chemical compositions are Ta, O, and N. The maximum average roughness of the coatings was prepared by pure argon sputtering. It is proved that the introduction of N_2_ reduces the surface roughness of the coatings and increases the surface hardness and elastic modulus of the coatings. Adhesive wear and brittle fracture are the two main wear forms of coatings. The wear debris is mainly composed of columnar particles and a flake structure.

## 1. Introduction

Compared with fossil energy, the power generation conversion rate is low, with high pollution and non-renewable disadvantages. Because it is clean and renewable, photovoltaic power generation has become the new energy that is widely used in human life and industry. Photovoltaic power generation is considered to have great potential in the power industry and has become a hot topic at home and abroad in recent years [1]. Studies have shown that the global solar photovoltaic market has developed rapidly with a growth rate of 50% over the past decade [2,3]. Because the actual working environment of photovoltaic cells often appears as dust, rain, and other bad weather, in order to avoid damage to photovoltaic cell components, high transmittance glass is commonly used as the cover protection component part [4,5]. Dust and gravel particles generate frictional wear when they accumulate on the surface of the glass cover due to wind factors, and this situation not only reduces the light transmission of the glass cover [6], but also affects the photovoltaic cell’s photoelectric conversion efficiency [7,8]. It is reported that in the Saudi region, within a few months, due to dust deposition, photovoltaic cell system power generation efficiency was reduced by more than 30% [9]. Many researchers have carried out a lot of research on the problem of friction and the wear of glass cover plates caused by dust accumulation. It has become a common method to apply functional coating on equipment surfaces in recent years, such as transparent coating [10], conductive coating [11], wear-resistant coating [12,13], and lubricating coating [14,15].

Transition metal nitride coatings are used as multifunctional coatings in many fields due to their high hardness, high wear resistance, chemical inertness, and high temperature stability. TaN has attracted increasing attention due to its excellent chemical and physical properties. It is widely used as a hard coating, wear-resistant coating, and integrated circuit diffusion layer [16]. Much of the research literature has focused on physical vapor deposition (PVD) technology, with pure Ta as a target, after the introduction of N_2_ and a tantalum ion reaction to form the TaN coating. CHEN R et al. used DC magnetron sputtering technology, with pure tantalum as a target, and found that a columnar structure exists in a single layer TaN coating [17]. A multilayer TaN coating has a higher hardness and lower friction coefficient than a single layer TaN coating. SEMPAH I et al. found that grain refinement by boron addition improves the hardness of the TaN coating, and the friction coefficient decreases with the increase in boron content [18]. SERNA-MANRIQUE M D et al. sputtered TaN coatings on 304 stainless steel items in a N_2_/Ar atmosphere [19]. It was found that the power has a direct relationship with the formation of specific grains in the coating, which further affects the corrosion resistance of the coating.

Ta_2_O_5_ coatings are often used as high-performance materials in optical devices [20] due to their good optical properties, thermal stability [21] and chemical stability [22] in the visible and near-infrared regions, and high refractive index [23]. Ta_2_O_5_ also has good mechanical properties such as wear resistance [24] and high hardness [25], and can be used as a protective coating. According to the relevant literature, Ta metal oxide Ta_2_O_5_ was proposed as the target.

In our previous study, the optical properties of Ta_2_O_5_ coatings prepared under sputtering powers of 100 W, 120 W, 140 W, 160 W, and 180 W were studied. The results showed that the transmittance of the coatings was high when the sputtering power was 140 W, indicating that the morphology was uniform and smooth. In order to further improve the mechanical properties of the coatings prepared under this sputtering power condition, N_2_ was introduced during the preparation process to study the effect of the N_2_ flow rate on the coating’s quality. In this paper, the mechanical properties and friction and wear properties of coatings under different nitrogen/argon ratios of 40Ar, 36Ar–4N_2_, 34Ar–6N_2_, and 32Ar–8N_2_ were studied. The purpose was to analyze the relationship between the sputtering gas flow rate and the mechanical properties of the coatings. In addition, the composition and roughness of the films were characterized.

## 2. Materials and Methods

The Ta_2_O_5_ thin films were prepared by RF magnetron sputtering technique through JGP045CA (Shenyang Scientific Instrument Co., Ltd. Xinyuan Street, Hunnan District, Shenyang, China) sputtering system [26]. The substrate was highly transparent quartz glass (10 mm × 10 mm × 2 mm) and N<111> type silicon wafer (7 mm × 7 mm × 0.5 mm). The samples were ultrasonically cleaned with acetone, anhydrous ethanol, and deionized water for 15 min with SCIENTZ-450 (Xinzhi Freeze-drying Equipment Co., Ltd. Jiao Chuan Street, Zhenhai District, Ningbo, China) equipment before deposition, dried with compressed air, and prepared for use. The back-bottom vacuum was pumped to below 5 × 10^−4^ Pa, and the deposition pressure was 0.8 Pa. The total gas flow rate for deposition was kept constant at 40 sccm (10%, 15%, and 20% for N_2_/(N_2_+Ar) in the experiment, respectively). The target material was Ta_2_O_5_ of 99.99% purity (φ30 × 3 mm, Beijing Goodwill Metal Technology Development Co., Ltd. Dazhong Temple, Haidian District, Beijing, China). The deposition temperature was room temperature, the sputtering power was 140 W, and the substrate rotation speed was 10 rpm (no negative bias). A clean target surface was obtained by glow discharge pre-sputtering for 10 min before deposition, and the deposition time was 45 min.

**Figure 1:**
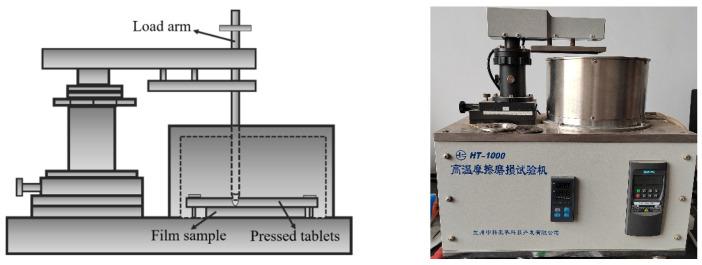
A ball on disc tribometer is used for wear and friction tests.

A ball on disc tribometer used for wear and friction tests was used to evaluate the friction and wear characteristics of the composite coating samples and ceramic balls at room temperature (HT-1000, Lanzhou Zhongke Kaihua Technology Development Co., Ltd. Nanchang Road, Chengguan District, Lanzhou, China). A ball on disc tribometer used for wear and friction tests is shown in Figure 1. Ceramic balls (φ5 mm) were pressed against the sample surface during sliding with a constant load of 2 N. The relative rotation of the sample and the ceramic was maintained at 100 rpm until the film on the sample surface failed. The microhardness (H) and Young’s modulus (E) of the sample film were tested by applying a load of 50 mn to the single crystal silicon wafer using a nanoindentation ZDT075-075 (Liansheng Technology Co., Ltd. Xiaolan Economic Development Zone, Jinsha Third Road, Nanchang, China) equipped with a diamond indenter. The nanoindentation depth should be less than 10% of the coating thickness. Scanning electron microscopy (SEM, Zeiss Sigma 300. Jena, Germany) was used to analyze the surface morphology and abrasion marks on the sample surface before and after the friction test, and to obtain an approximation of the film deposition thickness from the cross-sectional morphology of the coatings. Determination of film composition by energy spectrum of the sample surface was obtained by an energy dispersive spectrometer (Smart EDX). The surface morphology of the sample was obtained using an atomic force microscope (AFM, Bruker Dimension Icon, Ettlingen, Germany). At the same time, the surface roughness (Ra) and root mean square (RMS) of the samples were obtained.

## 3. Results

**Figure 2:**
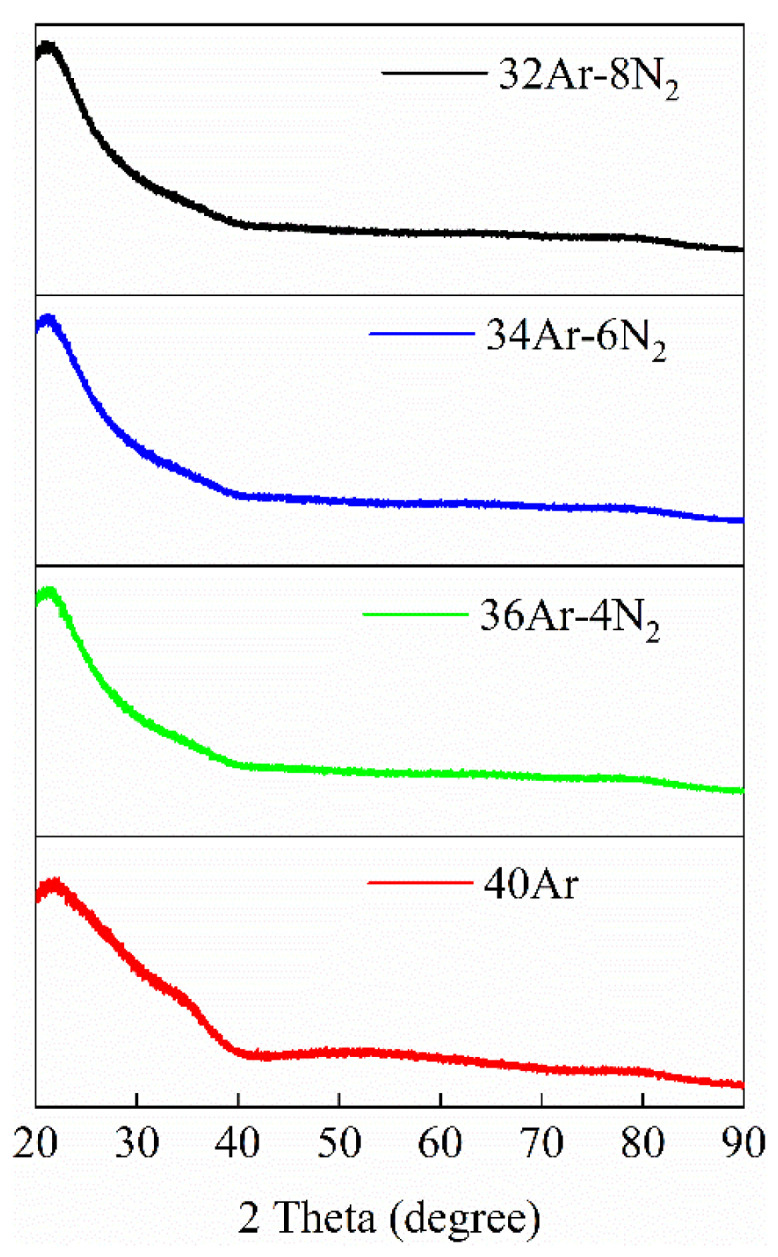
XRD spectra of Ta_2_O_5_ coatings deposited at sputtering power of 140 W with different N_2_ and Ar fluxes.

The XRD patterns of Ta_2_O_5_ coatings deposited under different flow rates of N_2_ and Ar are shown in Figure 2. XRD was used to analyze the phase structure of Ta metal composite coating deposited on the glass base substrate. It can be seen from Figure 2 that the coatings are amorphous when the sputtering gas conditions are 40Ar, 36Ar–4N_2_, 34Ar–6N_2_, and 32Ar–8N_2_, respectively. Because a large number of Ta atoms are released from the target, they are freely mixed with argon ion radicals and nitrogen ions in the chamber. One part of the Ta atom recombines with the oxygen atom to form a tantalum metal oxide, and the other part combines with the nitrogen atom to form a tantalum metal nitride. As a result, the dynamic energy of the Ta metal particles reaching the substrate is reduced, and the crystal structure cannot be formed by orderly arranging of the particle positions.

**Figure 3:**
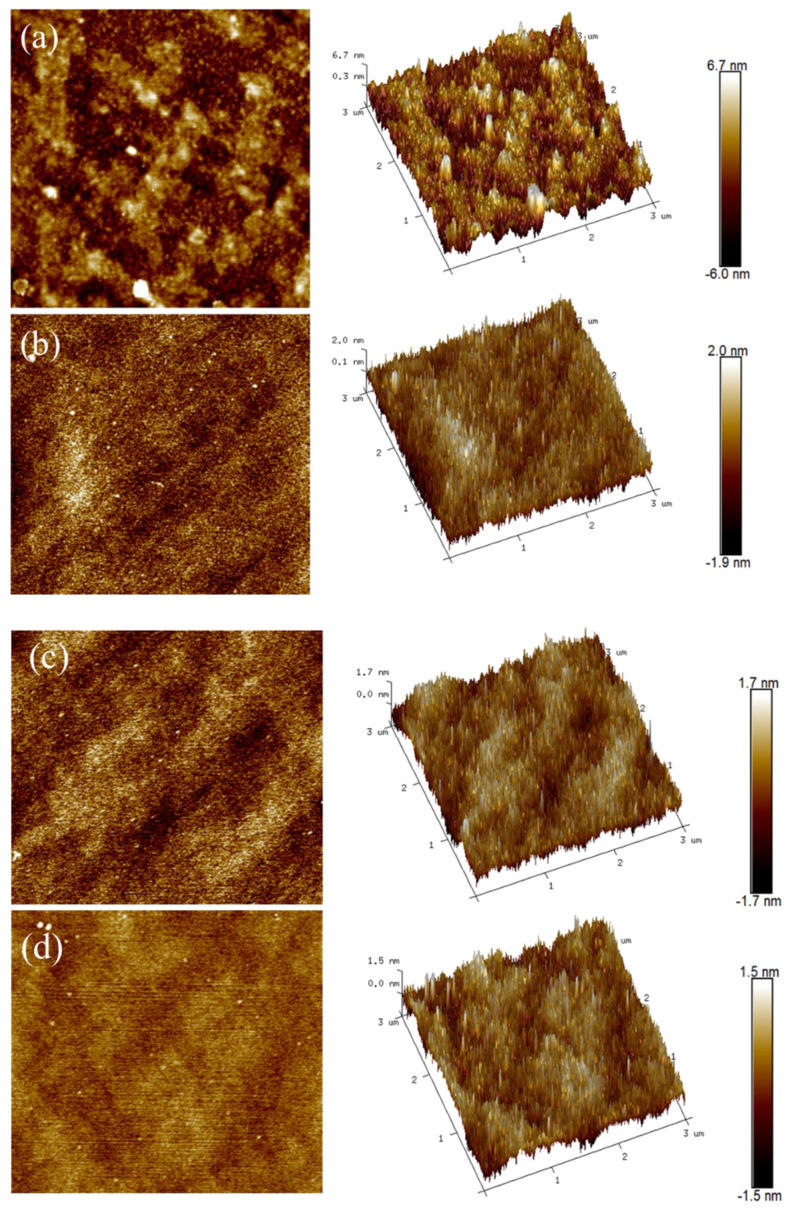
Surface roughness 2D and 3D AFM images of Ta_2_O_5_ coatings were prepared under different sputtering gas conditions. (**a**) 40Ar, (**b**) 36Ar–4N_2_, (**c**) 34Ar–6N_2_, (**d**) 32Ar–8N_2_.

Since the change in the N_2_ flow rate will affect the microstructure of the sample coating, the surface roughness of the sample was characterized by atomic force microscopy (AFM). The 2D and 3D morphology of the coatings’ surface roughness is shown in Figure 3. The scanning area is 3 μm × 3 μm. The coatings were prepared under pure argon sputtering conditions as shown in Figure 3a. The sputtered particles accumulate into a pyramid shape on the coatings’ surface, and there are broad peaks. The coatings prepared under mixed gas sputtering conditions are shown in Figure 3b–d. The sputtered particles on the surface of the coatings accumulate into the columnar structure. The peak decreases with the increase in the N_2_ flow rate. The maximum peaks on the surface of each coating are 6.7 nm, 2.0 nm, 1.7 nm, and 1.5 nm, respectively.

**Figure 4:**
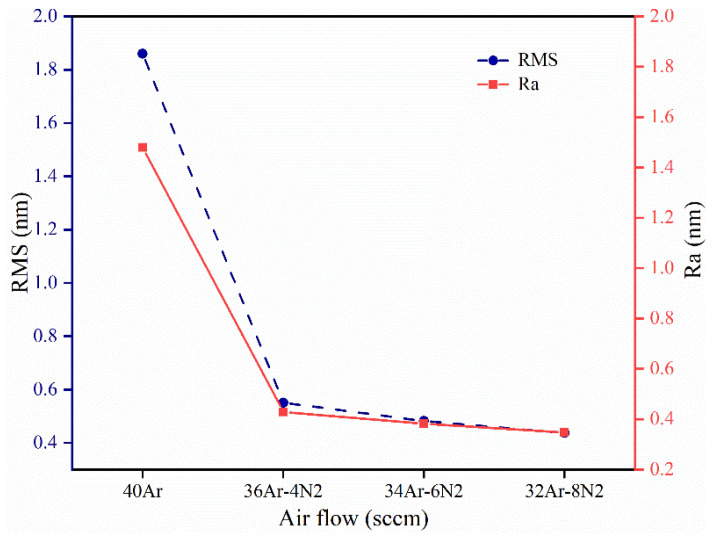
Surface Ra and RMS values of Ta_2_O_5_ coatings prepared under different sputtering gas conditions.

The roughness of coating samples prepared under different sputtering gas conditions was measured. The average roughness (Ra) and the root mean square (RMS) of the coatings’ surfaces are shown in Table 1 and Figure 4. The average roughness of the coating samples is nanoscale, indicating that the coatings’ surfaces are smooth. The mean values of Ra and RMS of the coating samples prepared under pure argon sputtering conditions are much larger than those of the coating samples prepared under mixed gas sputtering conditions. After N_2_ was introduced, the Ra value and RMS value of the coating surface decreased with the increase in the N_2_ flow rate.

**Figure 5:**
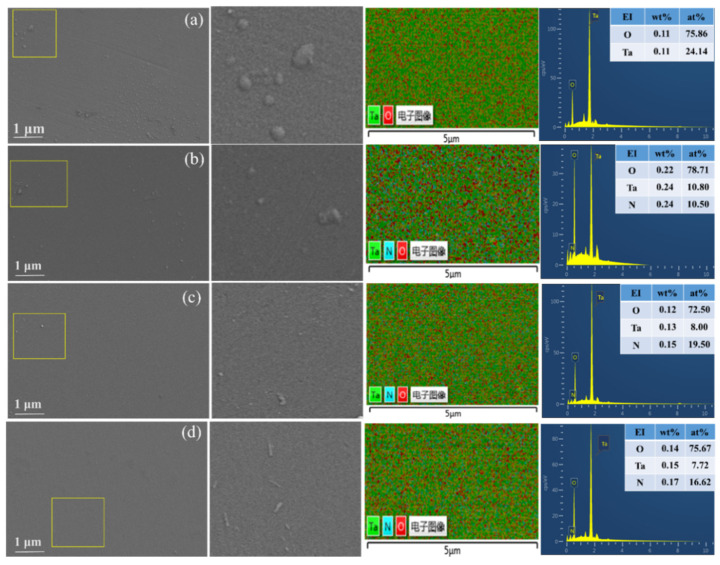
SEM images and EDS spectra of Ta_2_O_5_ coatings prepared under different sputtering gas conditions. (**a**) 40Ar, (**b**) 36Ar–4N_2_, (**c**) 34Ar–6N_2_, (**d**) 32Ar–8N_2_.

**Figure 6:**
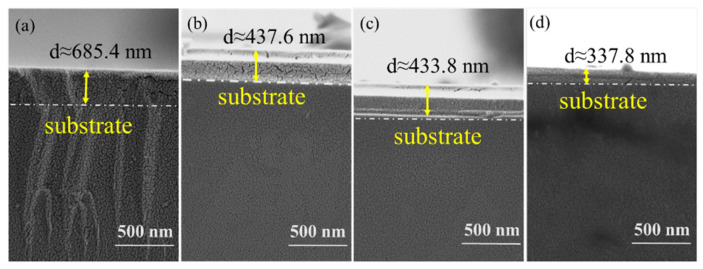
Cross-sectional SEM images of Ta_2_O_5_ coatings prepared under different sputtering gases. (**a**) 40Ar, (**b**) 36Ar–4N_2_, (**c**) 34Ar–6N_2_, (**d**) 32Ar–8N_2_.

In order to observe the surface morphology and element distribution of the coating sample after N_2_ was introduced, the SEM and EDS spectra of the Ta_2_O_5_ coating prepared under different sputtering gas conditions are shown in Figure 5. Figure 5a shows that there is a large area of the convex part formed by the accumulation of sputtered particles on the surface of the coating, and the size of the convex part is larger. Figure 5b–d show that only a few bulges were formed by the accumulation of sputtered particles on the coating surface. The changing trend of the surface morphology and roughness of the coatings are basically consistent with those of Figure 3 and Figure 4 and Table 1. The AFM and SEM surface morphology analysis of the coatings shows that the passage of N_2_ can change the sputtered particles’ stacking structure and optimize the surface quality of the coatings. The deposition thickness of the coatings was 685.4 nm, 437.6 nm, 433.8 nm, and 337.8 nm after SEM scanning of the cross-section of the coatings when the sputtering gas flow rate was 40Ar, 36Ar–4N_2_, 34Ar–6N_2_, and 32Ar–8N_2_. Cross-sectional SEM images of the Ta_2_O_5_ coatings prepared under different sputtering gases are shown in Figure 6.

The average concentration and distribution of elements in the coating samples were obtained by EDS, as shown in the table and energy spectrum in Figure 5a–d. The table in Figure 5a shows no nitrogen atoms in the coatings. The table in Figure 5b–d shows a gradual increase in the content of nitrogen atoms, consistent with changing sputtering gas conditions during preparation. The EDS spectrum showed that Ta, O, and N elements were evenly distributed in the coatings.

Hardness (H) and elastic modulus (E) are two important mechanical properties of materials. The ratio of H/E and H^3^/E^2^ is a parameter reflecting the ability of materials to resist plastic deformation, which is usually used to evaluate the toughness and wear resistance of materials [27,28]. The surface hardness and elastic modulus of Ta_2_O_5_ coatings prepared under different sputtering gas conditions are shown in Table 2. It can be seen from the data in the table that the hardness and elastic modulus of the coating samples prepared under pure argon sputtering conditions are smaller than those prepared under mixed gas sputtering conditions. The effect of the nitrogen–argon gas flow ratio on surface hardness and the elastic modulus of coatings showed that they both increased with increasing nitrogen flow. The increase in the nitrogen flow rate makes more nitrogen ions combine with tantalum ions to form a thicker nitride coating.

**Figure 7:**
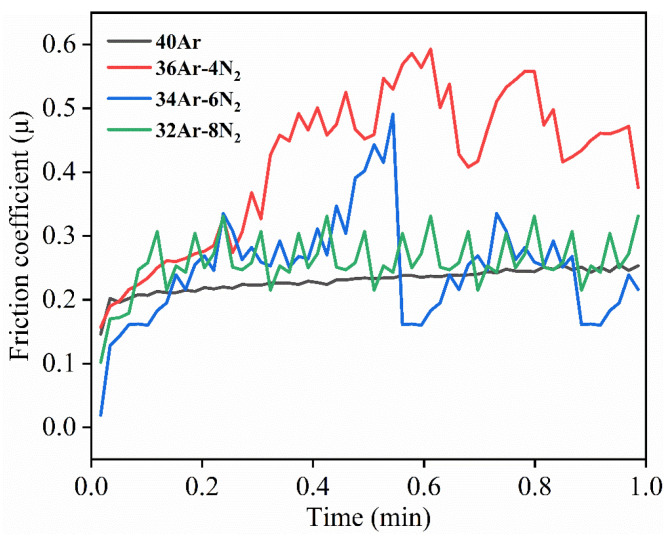
Friction coefficient of Ta_2_O_5_ coatings prepared under different sputtering gas conditions.

The friction coefficient curves of the Ta_2_O_5_ coatings prepared under different sputtering gases are shown in Figure 7. The fluctuation of the friction curve of the coatings prepared at a sputtering gas flow rate of 40Ar is the smallest. The fluctuation range of the friction coefficient curve of the coating samples is the largest when the sputtering gas flow rate is 36Ar–4N_2_. The fluctuations of the friction curves of the coating samples prepared at sputtering gas flow rates of 34Ar–6N_2_ and 32Ar–8N_2_ are relatively small. The average friction coefficient curves of the coatings were: 0.230, 0.415, 0.249, and 0.258.

**Figure 8:**
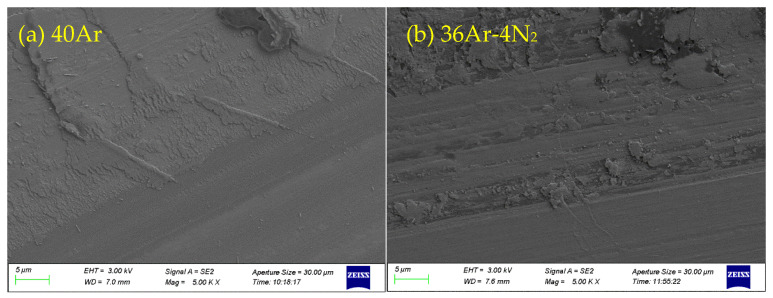

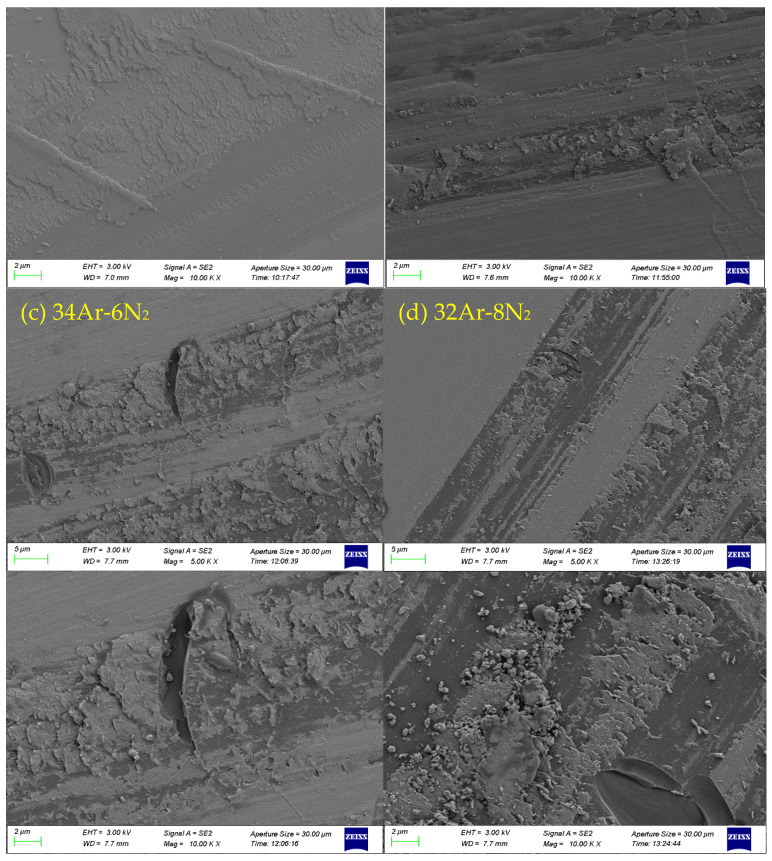
SEM images of wear scar morphology of Ta_2_O_5_ coatings prepared under different sputtering gas conditions. (**a**) 40Ar, (**b**) 36Ar–4N_2_, (**c**) 34Ar–6N_2_, (**d**) 32Ar–8N_2_.

In order to understand the main reasons affecting the friction coefficient of the coating samples and explore the friction and wear mechanism of the coatings, the coatings’ wear marks and debris were emphatically analyzed. The SEM images of the wear scar morphology of Ta_2_O_5_ coating samples prepared under different sputtering gas flow rates (magnification of 5 K and 10 K) are shown in Figure 8. Figure 8a shows that there is no spalling and brittle fracture in the wear scar when the sputtering gas is 40Ar, and a large area of the coatings transfer layer appears on the surface of the wear scar. Figure 8b shows that a small amount of the coatings on the surface of the wear scar peels off, transfers, and then restacks to appear as bulges when the sputtering gas is 36Ar–4N_2_, which is also the main reason for the large fluctuation in the friction curve. Figure 8c shows that the wear track exhibits brittle fracture and spalling when the sputtering gas is 34Ar–6N_2_. The crack may be the position where the friction curve of the coating fluctuates the most. There is no large area accumulation bulge in coating transfer, which is basically consistent with the fluctuation in the friction curve. Figure 8d shows that a large area of spalling appears on the wear scar when the sputtering gas is 32Ar–8N_2_.

**Figure 9:**
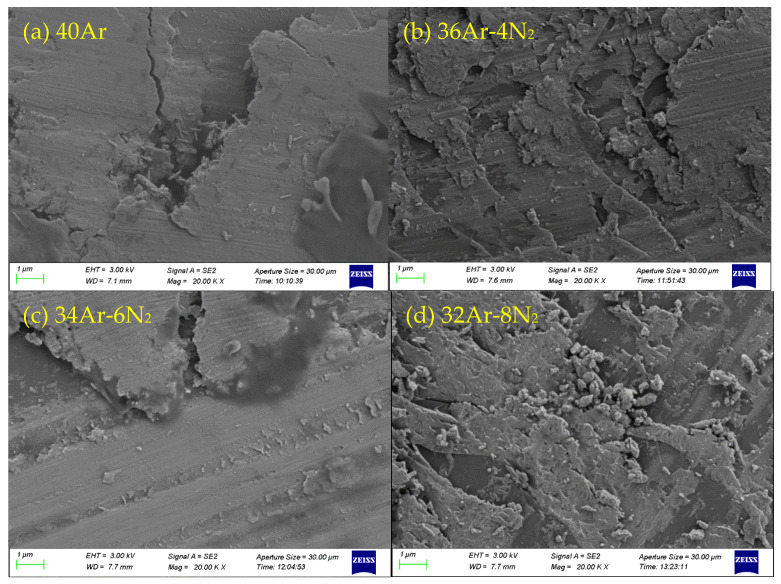
Wear debris morphology of Ta_2_O_5_ coatings prepared under different sputtering gas conditions. (**a**) 40Ar, (**b**) 36Ar–4N_2_, (**c**) 34Ar–6N_2_, (**d**) 32Ar–8N_2_.

Local amplification of the wear track (magnification 20 K). The wear debris morphology in the wear track is shown in Figure 9. It can be seen that the wear debris of the coating samples prepared under different sputtering gas conditions is very different. Figure 9a shows that the wear debris is mainly composed of fine columnar particles when the sputtering gas is 40Ar. Figure 9b shows that the wear debris is mainly composed of blocks and flakes when the sputtering gas is 36Ar–4N_2_. Figure 9c shows that the wear debris is mainly composed of columnar particles and flakes when the sputtering gas is 34Ar–6N_2_. Figure 9d shows that the wear debris is mainly composed of larger particles when the sputtering gas is 32Ar–8N_2_.

## 4. Conclusions

Ta_2_O_5_ coatings were prepared by RF magnetron sputtering under different sputtering gas conditions. The microstructure and mechanical properties of the coatings were evaluated by XRD, AFM, SEM, EDX, a nanoindenter, and a friction and wear tester. The results show that the prepared coating samples are amorphous film. The coating samples are mainly composed of the three elements, Ta, N, and O, and these are evenly distributed in the coatings. The coatings’ roughness decreases with the increase in N_2_ flow rate. The hardness and modulus of elasticity increase with increasing N_2_ flow. Friction and wear experiments show that the coatings’ wear is mainly adhesive wear and brittle fracture. The wear debris is composed of columnar particles and a flake structure. Comprehensive analysis shows that the introduction of nitrogen can reduce the coatings’ roughness and improve the mechanical and tribological properties of the coatings.

## Figures and Tables

**Table 1:** The average surface roughness of Ta_2_O_5_ coatings prepared under different sputtering gas conditions was measured by AFM.

Sample	40Ar	36Ar–4N_2_	34Ar–6N_2_	32Ar–8N_2_
Ra (nm)	1.48	0.429	0.382	0.347
RMS (nm)	1.86	0.551	0.482	0.437

**Table 2:** Surface hardness and elastic modulus of Ta_2_O_5_ coatings were prepared under different sputtering gas conditions.

Sample	Hardness GPa	Young’s Modulus GPa	H/E	H^3^/E^2^
40Ar	8.463	106.7	0.079	0.0540
36Ar–4N_2_	12.371	108.1	0.114	0.1620
36Ar–6N_2_	14.401	112.4	0.128	0.2363
32Ar–8N_2_	15.207	117.8	0.129	0.2534

## Data Availability

Not applicable.
